# Functional Antithrombin (AT) Deficiency Presenting as Multiterritorial Splanchnic Vein Thrombosis: A Case Report

**DOI:** 10.1155/crh/9938304

**Published:** 2026-10-05

**Authors:** Martha L. Cuervo, Angela M. Castañeda, Jaime A. Pineda, Valentina Montiel, Jheremy E. Reyes

**Affiliations:** ^1^ Department of Hematology Clínica Los Cobos Medical Center Bogotá Colombia; ^2^ Department of Hematology Los Cobos Medical Center Bogotá Colombia; ^3^ Department of Internal Medicine Clínica Los Cobos Medical Center Bogotá Colombia; ^4^ Department of Hematology Los Cobos Medical Center El Bosque University Bogotá Colombia

**Keywords:** anticoagulants, antithrombin deficiency, genetic variant, next-generation sequencing, portal vein, thrombophilia, venous thrombosis

## Abstract

Functional antithrombin (AT) deficiency is an inherited thrombophilic disorder that can present as venous thrombosis at atypical sites. We report the case of a 47‐year‐old man with a family history of venous thromboembolism who presented with thrombosis involving the splenoportal confluence and the superior mesenteric vein. Initial laboratory evaluation revealed normal AT activity levels and a negative standard thrombophilia panel. Due to the unusual thrombotic location and family history, extended genetic testing was performed, which identified a pathogenic heterozygous variant in *SERPINC1* NM_000488.3:c.749C > A (p.Thr250Asn), consistent with functional AT deficiency. After the resolution of gastrointestinal bleeding complications, long‐term anticoagulation therapy was reintroduced. This case underscores the importance of considering hereditary thrombophilia in patients with thrombosis at unusual sites even when standard laboratory studies are unremarkable.

## 1. Introduction

Antithrombin (AT) is a glycoprotein synthesized in the liver that belongs to the serpin (serine protease inhibitor) family and is encoded by the *SERPINC1* gene. It plays a crucial role in regulating the coagulation cascade through the inhibition of thrombin, factor IXa, and factor Xa, as well as by blocking platelet activation. Additionally, AT is involved in anti‐inflammatory mechanisms and contributes to the maintenance of the endothelial barrier integrity [1–4]. Structurally, it contains two key domains: the reactive center loop (arginine‐based) and the heparin‐binding site (HBS) [1].

AT deficiency is an autosomal dominant disorder caused by genetic variants in *SERPINC1*, which leads to impaired synthesis, structural abnormalities, and conformational instability of the protein, ultimately affecting its anticoagulant function [1, 5, 6]. The estimated prevalence in the general population ranges from 0.02% to 0.2% [5]. The most common clinical manifestation is deep vein thrombosis (DVT) [1, 2]. However, an increasing number of reports have documented thrombosis in unusual locations, such as the mesenteric, caval, renal, and retinal veins. Studies and case series suggest a higher prevalence of hereditary thrombophilia in patients with venous thromboembolism (VTE), including mesenteric vein thrombosis (MVT), estimated to be between 36% and 55% [7], highlighting the importance of thorough evaluation in MVT cases with no apparent risk factors.

Lane et al. classified AT deficiency into Type I (quantitative), with parallel reductions in AT activity and antigen levels, and Type II (qualitative), in which a structurally abnormal protein is produced despite a normal or near‐normal antigen level [6, 8]. Type II is further subdivided according to the functional domain affected: reactive site (RS) variants, which impair inhibition of thrombin and other target serine proteases rather than thrombin alone; HBS variants, which selectively reduce heparin cofactor activity while sparing progressive, heparin‐independent inhibition; and pleiotropic effect (PE) variants, which affect both domains and behave clinically like Type I [6, 8]. A transient, acquired form of AT deficiency has also been recognized, in which gene–environment interactions or acute physiological states (e.g., disseminated intravascular coagulation, nephrotic syndrome, and extracorporeal circulation) transiently lower AT levels and may be associated with a higher incidence of arterial thrombosis [8, 9].

Conventional laboratory methods for detecting AT deficiency may fail to identify certain pathogenic variants, particularly those involving *SERPINC1* genetic variants [3]. This limitation may result in underdiagnosis. In this context, molecular approaches such as next‐generation sequencing (NGS) have emerged as valuable tools with higher sensitivity for detecting genetic variants responsible for AT deficiency [10].

We present a case of a patient who developed thrombosis at an atypical site despite a negative standard thrombophilia panel. NGS identified a pathogenic *SERPINC1* variant that confirmed a functional AT deficiency.

## 2. Case Presentation

We report the case of a 47‐year‐old male originally from Colombia. He is a systems engineer with no relevant personal medical history but with a family history of recurrent unprovoked VTE disease in his sister, who had not undergone prior thrombophilia testing. He presented with severe diffuse abdominal pain accompanied by abdominal distension and emesis. Contrast‐enhanced abdominal CT revealed mesenteric venous thrombosis with extension into the splenoportal confluence and the superior mesenteric vein without signs of intestinal compromise or critical ischemia at that time (Figure 1). Given these findings, full anticoagulation with unfractionated heparin was initiated, and surgical intervention was initially deferred.

**FIGURE 1:**
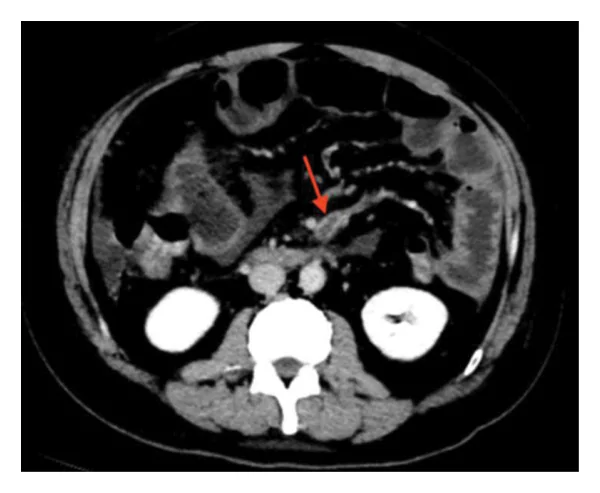
Axial contrast‐enhanced abdominal CT scan demonstrating a hypodense filling defect in the superior mesenteric vein, consistent with mesenteric venous thrombosis. Image was obtained from the patient’s medical record and used with patient consent.

However, owing to clinical deterioration and lack of improvement, the patient underwent exploratory laparotomy during which intestinal ischemia was identified, requiring bowel resection of 25 cm. Postoperatively, he was treated with enoxaparin (1 mg/kg every 12 h; anti‐Xa levels were not requested), which was later switched to a direct oral anticoagulant (DOAC).

During follow‐up, he developed upper gastrointestinal bleeding requiring transfusion; colonoscopy showed venous angiectasia versus varices in the right colon, and anticoagulation was temporarily withheld. A comprehensive thrombophilia workup—including an autoimmune panel, antiphospholipid antibodies, monoclonal protein screening, and protein C, protein S, and AT activity levels—was unremarkable, as were initial targeted molecular studies for the prothrombin G20210A and factor V Leiden variants. Given the absence of a diagnosis, the patient was referred to clinical genetics, where a NGS thrombophilia panel identified a heterozygous pathogenic *SERPINC1* NM_000488.3:c.749C > A (p.Thr250Asn) variant associated with hereditary thrombophilia due to functional AT deficiency was identified.

Considering the high risk of thrombotic recurrence, low‐dose anticoagulation was restarted indefinitely after resolution of the gastrointestinal bleeding. Family genetic testing was recommended to assess thrombotic risk in first‐degree relatives, as a fundamental pillar in the prevention of thrombotic events, high‐risk pregnancy management, and clinical decision‐making. However, genetic testing in family members could not be performed due to difficulties accessing the healthcare system, which precluded construction of a pedigree/genealogical tree at this time.

## 3. Discussion

### 3.1. Unusual Site Thrombosis

Thrombosis at unusual sites, whether arterial or venous, presents a significant diagnostic and therapeutic challenge for clinicians. Both primary and secondary care physicians may be required to counsel or manage asymptomatic individuals or patients with VTE who have a family history of thrombosis or hereditary thrombophilia [1]. Among these presentations, the splanchnic venous system—including the portal, splenic, and mesenteric veins—is the most frequently affected, with portal vein thrombosis (PVT) observed in up to 77% of reported cases; approximately 38% of patients show involvement of more than one splanchnic vessel [11]. These presentations often lack an overt provoking factor and warrant an individualized diagnostic workup, particularly when a family history of thrombosis is present [11, 12].

### 3.2. Functional AT Deficiency: Clinical and Molecular Correlation

Functional AT deficiency is a rare autosomal dominant disorder first described by Egeberg in 1965, who linked low plasma AT activity to familial thrombosis [4]. The estimated prevalence in the general population is 0.02%–0.2%, rising to 1%–5% among individuals with VTE. More than half of events occur before the age of 50 years, and roughly 40% are associated with a transient risk factor [4, 5]. Among inherited thrombophilic disorders, AT deficiency confers the highest thrombotic risk in both adults and children [4]. More than 300 genetic variants have been described in *SERPINC1*, accounting for the majority of hereditary AT deficiency [3].

In the presented case, the patient had a first‐degree relative with unprovoked thrombotic disease, a significant risk factor. Unlike most reported AT deficiency cases, which typically involve lower‐limb or pulmonary thrombosis [5, 13], our patient developed thrombosis across all three splanchnic territories. The identified variant, *SERPINC1* NM_000488.3:c.749C > A (p.Thr250Asn), does not correspond to a residue within the canonical RS (∼p.Arg425) or HBS (∼p.Arg24–Arg47) and, to our knowledge, has not been previously reported in ClinVar or the peer‐reviewed literature. Notably, both AT activity and AT antigen levels were within normal limits in this patient, precluding classification as a conventional Type I or Type II defect by routine laboratory criteria. This pattern parallels recently described *SERPINC1* variants that escape detection by standard functional and antigenic assays—for example, due to altered N‐glycosylation—and are identified only through molecular sequencing [3]. It underscores that a normal AT activity and antigen level does not exclude hereditary AT deficiency and that molecular testing should be pursued when clinical suspicion is high despite unremarkable conventional assays. While most thrombotic events in AT deficiency reflect quantitative (Type I) defects, mesenteric and cerebral venous sinus thromboses have also been described with qualitative (Type II) variants (Table 1) [5, 6, 13, 14].

**TABLE 1:** Classification of antithrombin (AT) deficiency and associated clinical implications.

Type	Mechanism	Laboratory findings	Clinical implications	Management considerations
Type I (quantitative)	Reduced synthesis and stability of AT	↓ activity and ↓ antigen levels	High risk of VTE, often at a young age	Long‐term anticoagulation; screening of family members
Type II—reactive site (RS)	Variant impairs the reactive center loop, reducing inhibition of thrombin AND other target serine proteases (e.g., factor Xa)	↓ activity with normal antigen level	Thrombotic risk similar to Type I	May be missed on basic assays; consider NGS
Type II—heparin‐binding site (HBS)	Variant reduces heparin cofactor activity	↓ activity with normal antigen level	Generally lower thrombotic risk than Type I/RS	Reduced heparin responsiveness; DOACs may be preferred
Type II—pleiotropic effect (PE)	Variant affects both the reactive site and the heparin‐binding site	↓ activity with normal antigen level	Variable risk, often similar to Type I	Requires genetic confirmation; individualize management
Transient AT deficiency	Acquired (e.g., DIC, nephrotic syndrome, ECMO)	↓ activity and/or ↓ antigen (reversible)	Risk varies with clinical context	Treat the underlying cause; consider temporary anticoagulation if needed

*Note:* Adapted from Pabinger and Thaler (Blood, 2019) and Bauer et al. (Ann Pharmacother, 2016), with permission.

### 3.3. Anticoagulation Management and the Bleeding Complication

Because the anticoagulant effect of low‐molecular‐weight heparin (LMWH), predominantly mediated through anti–factor Xa activity, is also AT‐dependent, its efficacy may be attenuated in patients with AT deficiency, and standard weight‐based dosing does not account for this variability. In this patient, enoxaparin was dosed at 1 mg/kg every 12 h in the early postoperative period, shortly after bowel resection—a setting already associated with elevated bleeding risk—but anti‐Xa levels were not measured, precluding confirmation that a safe and effective anticoagulant effect had actually been achieved. This absence of therapeutic drug monitoring, together with the reduced AT‐dependent catalytic efficiency inherent to this diagnosis, may have contributed to an unpredictable anticoagulant response and to the subsequent gastrointestinal bleeding. Routine anti‐Xa monitoring, particularly in the early postoperative period, may help individualize LMWH dosing and reduce this risk in patients with confirmed or suspected AT deficiency.

Current ASH guidelines recommend indefinite anticoagulation after unprovoked thrombosis, given an estimated recurrence risk of 7.4% per year [15, 16]. Registry and cohort data—including the START2 registry (4886 patients, 6.3% with AT deficiency) [16], a cohort of 45 patients with severe thrombophilia (11.2% with AT deficiency) [17], a cohort of 597 patients with inherited thrombophilia (1.5% with AT deficiency) [18], and a cohort of 57 patients with severe thrombophilia (18 with AT deficiency) [19]—consistently show that DOACs are not inferior to conventional therapy for thrombotic recurrence, although minor bleeding rates vary by agent. A meta‐analysis of seven randomized trials likewise found no difference in recurrence or major bleeding among thrombophilic patients treated with DOACs [20]. After an initial three‐to‐six‐month treatment phase, dose‐reduced DOACs (e.g., apixaban 2.5 mg twice daily or rivaroxaban 10 mg once daily) may be considered for secondary prevention [15].

## 4. Conclusion

Functional AT deficiency can present as thrombosis at unusual, multiterritorial sites and may evade detection by standard thrombophilia testing. Molecular testing was essential in establishing this diagnosis and should be considered in patients with atypical thrombotic presentations, particularly when standard laboratory assays are unremarkable. Cascade testing of first‐degree relatives is warranted to guide thrombotic risk stratification and family counseling; in this case, however, such testing could not be completed due to limited access to the healthcare system, underscoring a broader barrier to comprehensive thrombophilia care in similar settings.

## Author Contributions

All authors contributed equally and interchangeably to the conception and design of the study, data acquisition, drafting of the manuscript, critical revision of the content, and supervision of the work.

## Funding

This work received no external funding. No financial support was received from any organization for the submitted work.

All authors have declared that they have no financial relationships at present or within the previous 3 years with any organizations that might have an interest in the submitted work.

In compliance with the ICMJE uniform disclosure form, all authors declare the following: Payment/services info: All authors have declared that no financial support was received from any organization for the submitted work.

## Disclosure

All authors have declared that there are no other relationships or activities that could appear to have influenced the submitted work.

All authors have read and approved the final version of the manuscript. Martha L. Cuervo, the corresponding author, had full access to all data in this study and takes complete responsibility for the integrity of the data and the accuracy of the data analysis.

## Ethics Statement

Ethical approval for this case report was obtained from the Institutional Ethics Committee of the Clínica Los Cobos Medical Center (Bogotá, Colombia). Informed consent for treatment and open‐access publication of this case report was obtained from the patient.

All authors have confirmed that this study did not involve animal subjects or tissues.

## Consent

The patient allowed personal data processing, and informed consent was obtained from the patient included in the study.

## Conflicts of Interest

The authors declare no conflicts of interest.

## Data Availability

The data supporting the findings of this case report are available from the corresponding author upon reasonable request, with no confidentiality restrictions.
